# Thermal Energy Harvesting on the Bodily Surfaces of Arms and Legs through a Wearable Thermo-Electric Generator

**DOI:** 10.3390/s18061927

**Published:** 2018-06-13

**Authors:** Antonino Proto, Daniele Bibbo, Martin Cerny, David Vala, Vladimir Kasik, Lukas Peter, Silvia Conforto, Maurizio Schmid, Marek Penhaker

**Affiliations:** 1 Department of Cybernetics and Biomedical Engineering, VŠB-Technical University of Ostrava, 17. listopadu 15, 70833 Ostrava-Poruba, Czech Republic; antonino.proto@vsb.cz (A.P.); martin.cerny@vsb.cz (M.C.); david.vala@vsb.cz (D.V.); vladimir.kasik@vsb.cz (V.K.); lukas.peter@vsb.cz (L.P.); 2 Department of Engineering, University of Roma Tre, Via Vito Volterra, 62, 00146 Rome, Italy; daniele.bibbo@uniroma3.it (D.B.); silvia.conforto@uniroma3.it (S.C.); maurizio.schmid@uniroma3.it (M.S.)

**Keywords:** body temperature, energy harvesting, human daily activities, thermoelectricity, wearable device

## Abstract

This work analyzes the results of measurements on thermal energy harvesting through a wearable Thermo-electric Generator (TEG) placed on the arms and legs. Four large skin areas were chosen as locations for the placement of the TEGs. In order to place the generator on the body, a special manufactured band guaranteed the proper contact between the skin and TEG. Preliminary measurements were performed to find out the value of the resistor load which maximizes the power output. Then, an experimental investigation was conducted for the measurement of harvested energy while users were performing daily activities, such as sitting, walking, jogging, and riding a bike. The generated power values were in the range from 5 to 50 μW. Moreover, a preliminary hypothesis based on the obtained results indicates the possibility to use TEGs on leg for the recognition of locomotion activities. It is due to the rather high and different biomechanical work, produced by the gastrocnemius muscle, while the user is walking rather than jogging or riding a bike. This result reflects a difference between temperatures associated with the performance of different activities.

## 1. Introduction

Nowadays, harvesting the energy on the human body is becoming a popular means to power wearable devices [1,2,3,4]. Wearables are increasingly being used in different health-related applications, thanks to the availability of miniaturized technologies. By integrating data processing into wearables, it is possible to capture a variety of variables associated with the health and safety of human beings [5,6,7,8]. Unfortunately, the battery size determines the operating time of wearables, thus limiting applicability for long-term monitoring [9,10].

Among all technologies used to harvest energy from environmental sources, recovering the energy associated to the heat produced by the human body is an interesting option from an energy perspective [11]. In the following section, first the aspects related to the physiological processes and the body heat exchange with the environment will be analyzed. Then, the previous solutions leveraging on the human heat energy harvesting will be reviewed. The Materials and Methods section shows the proposed solution, and obtained results will be analyzed in the Discussion section. Conclusions will complete the work.

### 1.1. Physiological and Environmental Aspects

Human body physiological parameters, and environmental conditions affect the amount of thermal energy that can be harvested from body surfaces [12]. The former include the position and the number of subcutaneous blood vessels, the characteristics of the living tissue (thickness of the fat layer, depth of the muscle tissue, and anatomy of the skin surface), and the physiological state of the body (metabolism, blood perfusion and sweat secretion). The Pennes’ bioheat equation includes all these parameters, and it has been used to characterize the influence of the blood flow on the body temperature distribution [13,14]. Regarding environmental conditions, which influence the heat transfer between living tissues, the main factors are the temperature and the humidity of the medium between two living tissues. 

In addition, body physiological states may change based on a number of situational variables, such as weather, and activities performed by people (e.g., working at a desk, as compared to performing sport activities): blood perfusion and metabolic heat generation significantly increase when a person is taking multiple physiological activities [15]. The most effective situation for harvesting the human heat energy is thus when the body is under an excited physiological state. 

From the above considerations, it is straightforward that skin temperature is non-uniform on the body surface: Table 1 shows the average values of skin temperatures measured at different body positions. Results are summarized based on the works proposed by Yang et al. [16], Zaproudina et al. [17] and Webb [18]. In the first two studies, body temperatures were measured by means of infrared thermography, while Webb collected data through multiple thermistor probes. Similar values of body temperatures are reported in other works [19,20].

From these data, it is clear that being under low environment temperatures leads to the most beneficial situations to harvest wasted human heat: a rise of about 10 °C in the air temperature produces an average increase of about 4 °C for the skin temperature.

### 1.2. Thermoelectric Generators

In 1821, Seebeck discovered thermoelectricity [21]. It can be summarized by saying that a voltage difference across two dissimilar metals or semiconductors appears in the presence of a temperature difference between them. Thus, in the middle of the 20th century, scientists around the world deepened the study on semiconductors as thermoelectric elements, thus creating the first Thermo-electric Generator (TEG) [22]. A TEG consists of multiple pairs of p- and n-type elements that are electrically connected in series by two metal conductors. In addition, two ceramic plates encapsulate the thermoelectric elements for their electrical insulation, but making the TEG thermally conducting.

Therefore, by placing a TEG on the body surface, it is possible to harvest the electrical energy by exploiting the thermoelectricity, i.e., the Seebeck effect, which occurs due to the temperature difference between the two opposing sides of the TEG: the one in contact with the skin, and the one facing the environment.

Integration of TEGs into devices grew in the late nineties: the Seiko Company developed the first wristwatch powered by a TEG [23]. The TEG power output was approximately 22.5 µW. This amount of power could drive a watch and simultaneously recharge its battery, since the value of the power output was well in excess of the amount needed for powering the watch (1 µW). After that, many researchers designed autonomous devices: between 2004 and 2008 the IMEC group developed wearables powered by the human heat. At first, Leonov et al. [24] designed a watchstrap with a single layer TEG, made of 128 thermocouples connected in series: they studied the thermal features of the human body. Then, a 4-layer TEG with 5000 thermocouples was designed to power a conventional pulse oximeter [25]. This 4-layer TEG generated up to 200 µW when a temperature difference of about 8 °C occurred between its two sides. In addition, in 2008 the researchers developed a self-powered 2-channel electroencephalography system [26], by realizing a hot side area of approximately 64 cm^2^. The system could generate a power output of approximately 2.2 mW. Based on these, the forehead has been identified as the best position for harvesting human heat, since the forehead provides the largest heat flow on a quite large area. Again, in 2009, the same research group proposed the first shirt for harvesting body thermal energy while people are performing normal daily activities [27,28]: 14 TEG modules placed in the shirt generated up to 1 mW when the user was working at the desk, whereas while walking on a sunny day, the power output reached up to 2–3 mW.

For harvesting body thermal energy directly from the arm, Lossec et al. [29] proposed a system made by stacking two TEGs with a black heatsink on the cold surface. The black surface increased the emissivity of the cold side, and the coefficient value about the TEG heat transfer/radiation parameter. With a temperature difference of about 15 °C, the power output reached up to 7 µW/cm^2^ during rest, and 30 µW/cm^2^ while the user was walking. In addition, Voss et al. [30] placed a Velcro strap, with an integrated TEG, on the upper arm, to produce electrical energy while users were performing locomotion activities, such as walking and jogging. The values of power output reached up to 0.5 mW for a temperature difference of about 8.5 °C. In all of the aforementioned systems, the heatsink was placed on the cold side of the TEG for improving the thermal coupling between TEG and environment, thus resulting in an increase of the power output [31]. However, the heat sink placement hinders wearability, and it makes the device uncomfortable for the human daily use.

Nowadays, in the commercial market, a smartwatch, i.e., the Matrix PowerWatch, is fully powered by a TEG. The watch is able to provide accurate information about calorie count, step count, and sleep track. However, its price is still very high since it costs is approximately $250.

Anyway, despite the rather large amount of solutions presented in the literature, which prominently focus on the TEG placement on the upper body parts, to the authors’ knowledge there is a notable lack of studies targeting the placement of TEGs on the lower limbs. For this reason, the proposed work focuses on the comparative analysis of the power harvested on arm and leg, by means of a TEG, without making discomfort to the user. We would affirm that the TEG placement on legs is a promising and different way to design and develop new self-powered, wearable, devices.

## 2. Materials and Methods

### 2.1. Thermoelectric Effect

A TEG produces measurable electrical energy by exploiting the thermoelectric effect, i.e., Seebeck effect. A quantitative constant describes the Seebeck effect, and the following equation defines it, as follows:

(1) $$\alpha =-\frac{\Delta V}{\Delta T},$$

where ΔV is the electrical voltage difference, and ΔT is the temperature difference between the two dissimilar metals or semiconductors. α is measured in µV/K.

Thermoelectric figure of merit (ZT), denotes transduction efficiency value of thermoelectric materials. It is a dimensionless quantity, and the following equation defines it, as follows:

(2) $$\mathrm{ZT}=\frac{{\sigma \alpha }^{2}T}{\kappa },$$

where α is the Seebeck coefficient, T is the average temperature, and σ and κ are the electrical and thermal conductivity of materials, respectively. In order to define the working efficiency of TEG, the following equation combines the figure of merit ZT and the expression about the Carnot cycle ($\frac{T_{H}-T_{C}}{T_{H}}$). It is as follows:

(3) $$\eta =\frac{T_{H}-T_{C}}{T_{H}}\;\frac{\sqrt{1+\mathrm{ZT}}-1}{\sqrt{1+\mathrm{ZT}}+\frac{T_{C}}{T_{H}}},$$

where η can assume a value between zero and one. The ZT value is the most critical parameter for a TEG. In today’s best commercial TEGs, ZT is about 1 at 25 °C [32]. It means that TEGs operate at only 10% of the Carnot efficiency [33]. The 30% of Carnot efficiency, comparable to home refrigeration, could be reached by a TEG with a ZT equal to 4, but this value cannot be achieved with current TEG modules [34].

### 2.2. Thermoelectric Generator Chosen

In this study, we chose the TES1-12704 Peltier module as TEG. It is low-cost, and integrates a large number of couples of p- and n-type elements into a small area of 9 cm^2^. Table 2 lists the main features of the TES1-12704 module.

**Figure 1:**
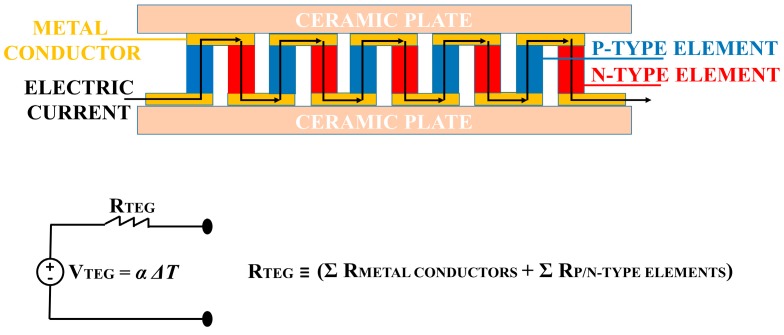
Structure of the TEG module and its equivalent, simplified electrical circuit.

Figure 1 shows the structure of the TEG module and its equivalent, simplified electrical circuit.

**Figure 2:**
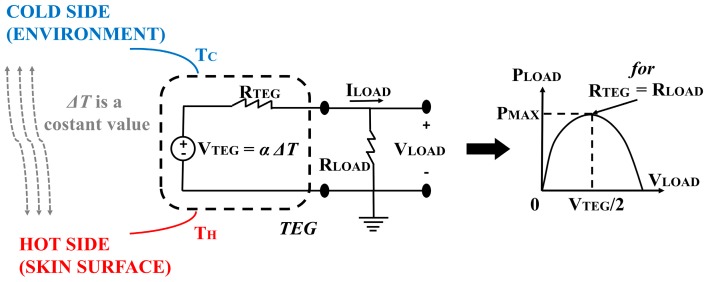
Electrical circuit for the measurements of power generated by the TEG, with the corresponding graph about the track of the power output while measuring the closed circuit voltage.

In Figure 1, a resistor (R_TEG_) in series with a voltage generator is the simplified electrical circuit for the TEG module. The TEG produces an electrical current flow in an external circuit if a temperature difference (ΔT) between its two sides is applied. The ΔT value determines the magnitude of the TEG voltage (V_TEG_) and the direction of the heat flow determines the voltage polarity. Furthermore, a ΔT change, across the TEG sides, causes a variation of the resistance value, R_TEG_ [35,36,37]. Thus, in the application field of human heat energy harvesting, it is difficult obtain a stable value for R_TEG_, since the temperature across the two sides of TEG changes over time, as the result of modifications of the physiological state of the body, and from the external unpredictable environmental conditions. Figure 2 shows the electrical circuit to measure the power generated by the TEG.

For a given temperature difference between the two sides of TEG, if the impedance matching is obtained (R_LOAD_ = R_TEG_), the maximum value of the power output (P_MAX_) is obtained for a voltage value (V_LOAD_) equals to the half of the open circuit voltage value (V_TEG_/2) [38]. The following equations describe the relation between V_LOAD_ and P_LOAD_:

(4) $$V_{\mathrm{LOAD}}=V_{\mathrm{TEG}}\frac{R_{\mathrm{LOAD}}}{R_{\mathrm{TEG}}+R_{\mathrm{LOAD}}}\Rightarrow P_{\mathrm{LOAD}}=\frac{V_{\mathrm{LOAD}}^{2}}{R_{\mathrm{LOAD}}},$$

(5) $$\frac{{\mathrm{dP}}_{\mathrm{LOAD}}}{{\mathrm{dR}}_{\mathrm{LOAD}}}=\frac{d\left(\frac{V_{\mathrm{LOAD}}^{2}}{R_{\mathrm{LOAD}}}\right)}{{\mathrm{dR}}_{\mathrm{LOAD}}}=0\Leftrightarrow R_{\mathrm{LOAD}}=R_{\mathrm{TEG}},$$

During the execution of the experiment, the values of the voltage output (V_LOAD_) were measured and acquired by the NI-USB-6210 data acquisition system (DAQ) (see Supplementary Materials, Figure S1).

### 2.3. System for Temperature Measurements

In order to acquire temperature data on both sides of the TEG, NTC 10K3MBD1 thermistor probes were connected to a multichannel recording system (see Supplementary Materials, Figure S2). The temperature sensor has a time response of about 400 ms, with a resolution value of ±0.2 °C in the temperature range from 0 °C to +70 °C. Two channels of the device were used to acquire the temperature data on both sides of the TEG, and a third one was used for the room temperature measurement. The proposed system relies on a 4-wire schematic configuration with a 100 µA current source based on REF200 integrated circuit. An OPA335 integrated circuit amplifies the measured signals of approximately 100 times, and an A/D converter with a sampling frequency of 10 samples/s converts them to have a display visualization. Temperature sensors were calibrated by means of a Fluke 1523 reference thermometer, which uses a Haart 5611T temperature sensor (resolution ± 0.01 °C, range 0–100 °C).

### 2.4. Fabric Band for Supporting the TEG

In order to place the TEG directly onto the skin, a fabric band made of PVC and gauze acted as a support for the TES1-12704 module. PVC fabric is a nonwoven fabric made up of a group of long fibers with a random pattern. It guarantees excellent thermal insulation. Therefore, it was chosen as the bottom layer of the band to thermally insulate the contact area between skin and TEG. 

Conversely, the gauze was used for the top layer of the band, as it is a structure with a loose open weave, a thin netting, with a good feature of breathability.

**Figure 3:**
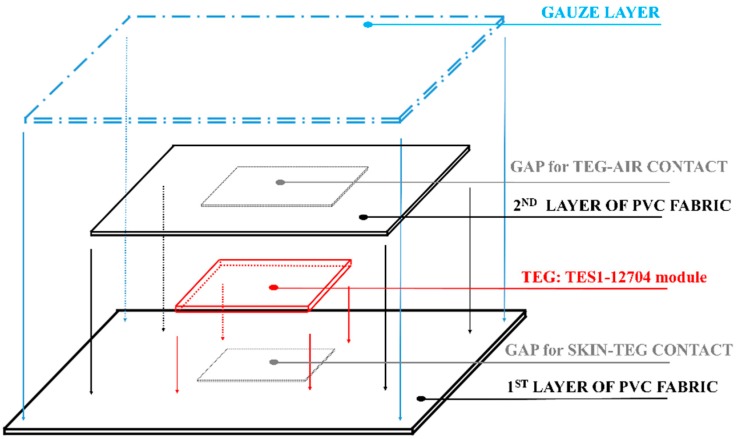
The manufacturing steps for the band development.

Figure 3 shows the manufacturing steps for developing the fabric band. At first, the PVC fabric was cut to form a rectangular sheet, with area size of approximately 350 cm^2^. A gap for the contact area between skin and TEG was made by cutting the PVC sheet in its central part (a square hole with area size of about 2.5 cm × 2.5 cm). Thus, the TEG, i.e., 3 cm × 3 cm, was encapsulated between the mentioned PVC sheet and a second PVC layer, which is also with a hole in its central part, but with a larger area size (2.9 cm × 2.9 cm). Finally, a transpiring layer, made of gauze was placed on the PVC fabric to enhance the stability of the system, but not to thermally isolate the TEG from the environment. All the fabric layers were knitted by using cotton yarns. To complete it, four laces were sewn on its ends. In the Supplementary Materials, Figures S3 and S4 show the chosen materials and the developed band, respectively. 

### 2.5. Human Body Positions for the Placement of TEG

The placement of the TEG on human body occurred on four body positions, two in the upper and two in the lower body parts. In order to improve the fit of the TEG onto the skin, the four different body parts were chosen with sufficiently large skin areas, and in correspondence to the superficial muscles of arm and leg. Biceps brachii for the arm anterior, and flexor carpi radialis for the forearm were the muscles corresponding to the placement of the TEG in the upper body part, while gracilis for the thigh and gastrocnemius for the calf were the chosen muscles for the leg (see Supplementary Materials, Figures S5 and S6). 

## 3. Experimental Section

We performed three experimental tests: (1) preliminary measurements; (2) execution of human daily activities in a controlled environment; (3) execution of human daily activities in a real scenario.

### 3.1. Preliminary Maesurements

Two healthy male volunteers stayed in resting position for more than 20 min, in which the voltage values were acquired in accordance to the following time-intervals: first minute, fifth minute, tenth minute, and twentieth minute.

These preliminary measurements were made to find out the value of the resistive load that maximizes the power output. To find out the optimum resistor load, the voltage data were acquired by varying the external load in the range: 1–32 Ω. Particularly, the range of resistor loads is as follows: 1.12 Ω, 3.88 Ω, 4.68 Ω, 5.71 Ω, 7.61 Ω and 31.84 Ω. The TEG was placed on each of the four aforementioned skin areas. 

During all the performed tests, a thermistor probe measured the room temperature, which was 25.0 °C. Moreover, two probes were connected to the top and bottom sides of the TEG to measure the temperature difference values.

**Figure 4:**
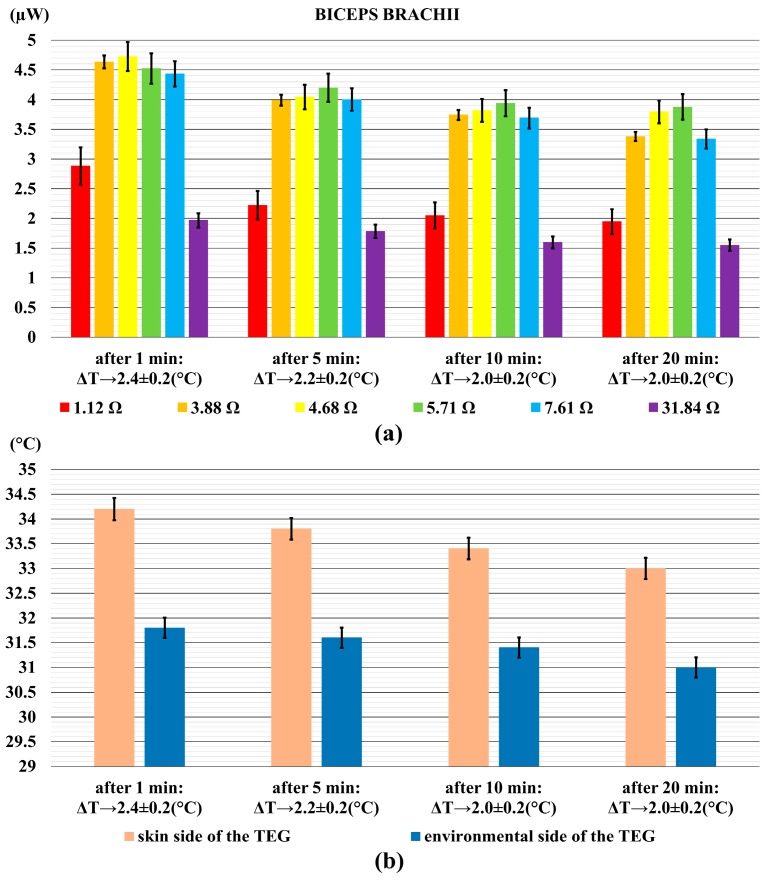
The power output generated by the TEG on the anterior arm anterior (biceps brachii) (**a**); and the relative temperature difference (**b**). Error bars represent standard deviations.

Figure 4a shows the power output generated by the TEG when it was placed on the arm anterior, whereas Figure 4b shows the relative temperature difference between the two sides of the TEG. In the diagram of Figure 4a the power output reached up to 5 µW after 1 min, and it decreased up to 4 µW at the end of the measurements. The discrepancy of approximately 1 μW is based on the decrease of the temperature difference. As it can be clearly seen in Figure 4b, the temperature difference decreases from 2.4 °C to around 2.0 °C.

**Figure 5:**
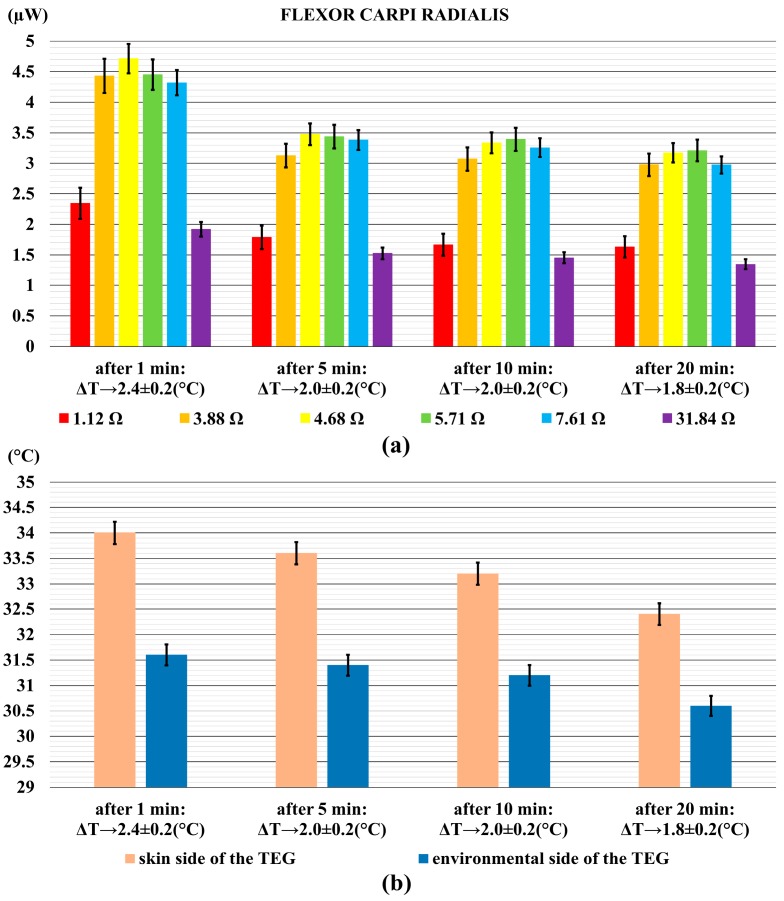
The power output generated by the TEG on the forearm, (flexor carpi radialis) (**a**); and the relative temperature difference (**b**). Error bars represent standard deviations.

Figure 5a shows the power output generated by the TEG when it was on the forearm, whereas Figure 5b shows the relative difference of temperatures between the skin and the environment. In the diagram of Figure 5a, the power output reached up to 4.8 µW after 1 min, and it decreased to 3.3 µW at the end of the measurement. Again, the variation of approximately 1.5 µW was in accordance to the decrease of temperature difference. As it can be clearly seen from Figure 5b, it decreased from 2.4 °C to 1.8 °C.

**Figure 6:**
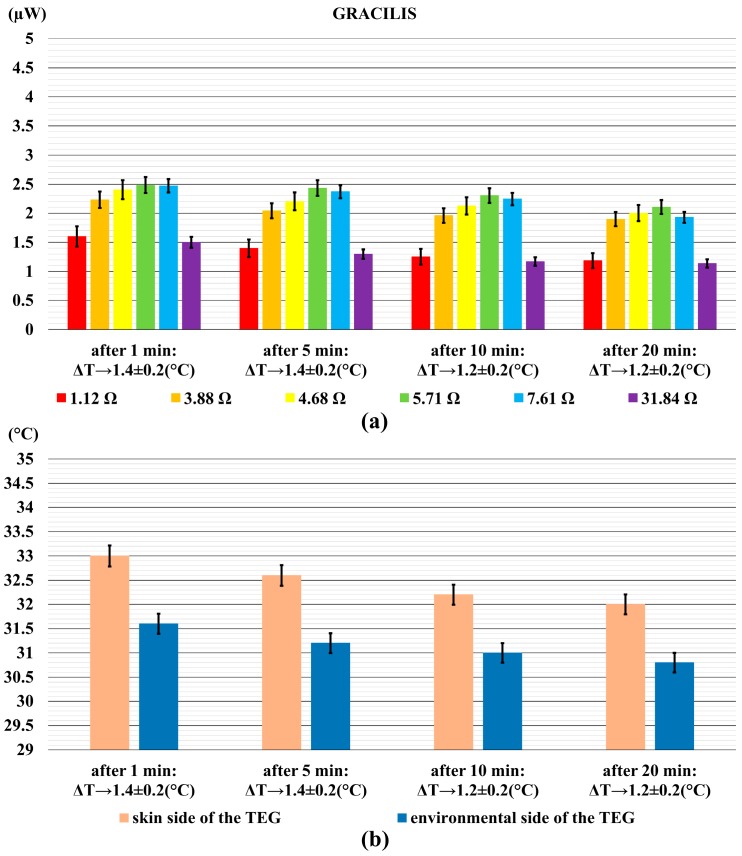
The power output generated by the TEG on the thigh, (gracilis muscle) (**a**); and the relative temperature difference (**b**). Error bars represent standard deviations.

Figure 6a shows the power output generated by the TEG when it was on the thigh, and Figure 6b shows the relative temperature differences between skin and environment. In this case, the power output reached up to 2.6 µW after 1 min, and decreased to 2.1 µW at the end of the measurement. It is depicted by the decrease of the temperature difference from 1.4 °C to 1.2 °C.

**Figure 7:**
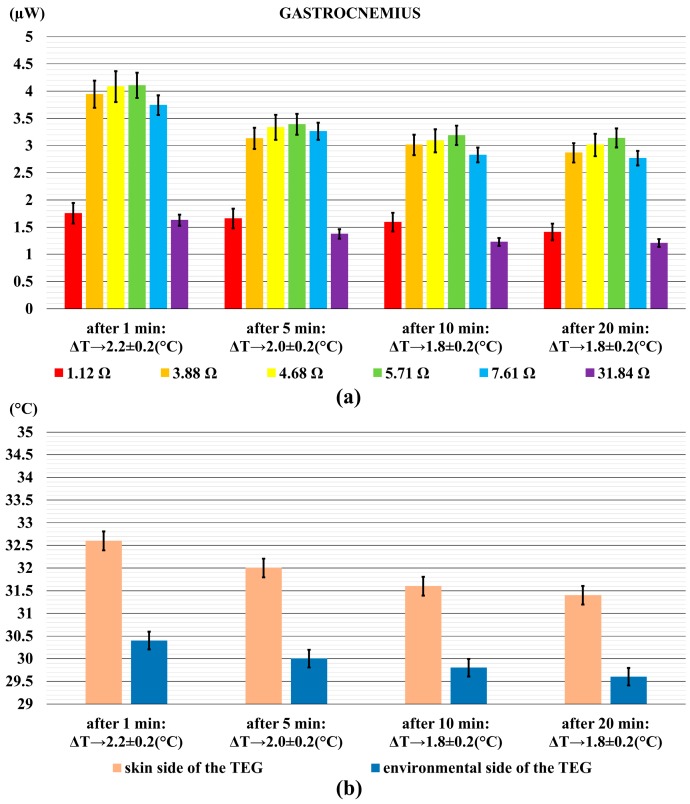
The power output generated by the TEG on the calf, (gastrocnemius muscle) (**a**); and the relative temperature difference (**b**). Error bars represent standard deviations.

A similar behaviour was found for the power output generated by the TEG placed on the calf (see Figure 7a): the power output reached up to 4.4 µW after 1 min, and it decreased to 3.35 µW at the end of the measurement. In this case, the decrease of the temperature difference was from 2.20 °C to 1.80 °C (see Figure 7b).

For all the performed measurements, results related to the best power values suggest a resistor load in the range from 4 to 6 Ω, as visible from the upper panels of Figure 4, Figure 5, Figure 6 and Figure 7.

### 3.2. Execution of Human Daily Activities in a Controlled Environment

Sitting, walking and jogging were the activities performed by users to measure the amount of electrical power generated by the TEG. We performed measurements in a controlled environment. Based on the results obtained in the previous experimental stage, the 5.71 Ω resistor load was used in the measurement circuit. 

Four healthy male volunteers (age: 25 ± 5 years; body weight: 69 ± 10 kg; height: 174 ± 6 cm) performed for three times the following cycle of multiple activities: sitting position (two minutes), walking (two minutes), again sitting position (two minutes), jogging (two minutes), again sitting position (two minutes). Each volunteer has repeated this cycle of multiple activities three times. During tests, the TEG was only placed on the biceps brachii and the gastrocnemius, because these body parts generated, in the preliminary measurements, the largest amount of power on arm and leg, respectively.

**Figure 8:**
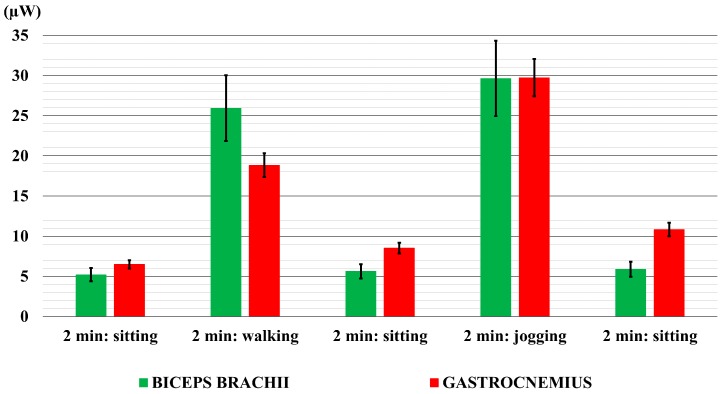
The mean values of the power output generated by the TEG on the calf, in correspondence to the gastrocnemius muscle (red bars), and on the arm anterior in correspondence to the biceps brachii muscle (green bars). Error bars represent standard deviation values.

Figure 8 shows the mean values of power output, with the standard deviation values. The measured temperature in the controlled environment was always around 23 °C. At first, it is important to affirm that the measured value of room temperature during the execution of these activities was 23 °C. It is 2 °C lower than the measured room temperature in the first stage of the experiments (25 °C). Therefore, in Figure 8 the power values related to the first two minutes of the sitting (5.5 µW for the biceps brachii and 6.5 µW for the gastrocnemius) resulted quite higher than the values in Figure 4a for the biceps brachii (4.5 µW), and in Figure 7a for the gastrocnemius (4 µW). 

Again, the power values generated by the TEG placed on the arm during all the three sitting periods were almost the same, (5.5 µW ± 0.3 µW). Conversely, the biomechanical work done by the gastrocnemius muscle during walking and jogging activities led to an increment of the power values for the last two sitting periods (8.5 µW ± 2.0 µW). 

As regards to the jogging activity, the power values for both the considered body parts are almost the same (30 µW). As regards to the walking activity, the placement of TEG on the biceps brachii produced higher power values (25 µW) than the values generated by placing the TEG on gastrocnemius (18 µW). 

In addition, the power generated on the biceps brachii is similar for both the walking and jogging activities. Conversely, the power generated by TEG on the calf, in correspondence of the gastrocnemius muscle, resulted quite different for walking and jogging, with a discrepancy of more than 10 µW. This is due to the increased biomechanical work of the gastrocnemius muscle while the user is performing these activities, which clearly increases in the jogging case. Conversely, the biomechanical work of the biceps brachii muscle is approximately the same for both the walking and jogging activities.

Once again, regarding walking and jogging, the values of the standard deviation for the measurements with the TEG placed on the arm are higher than those in which the TEG was on the calf. This result denotes that people move their upper limbs with higher inter-individual variability than lower limbs.

### 3.3. Execution of Human Daily Activities in Real Scenarios 

**Figure 9:**
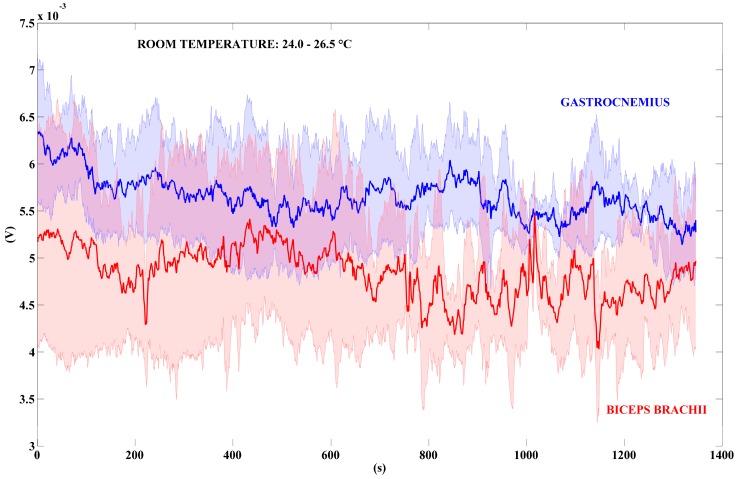
Voltage output signals generated by the TEG. The bold red line represents the mean value of the TEG placed on the biceps brachii; the bold blue line refers to the gastrocnemius. The blue and red areas around the bold lines represent the standard deviations (n = 5). Time-interval: 1350 s.

**Figure 10:**
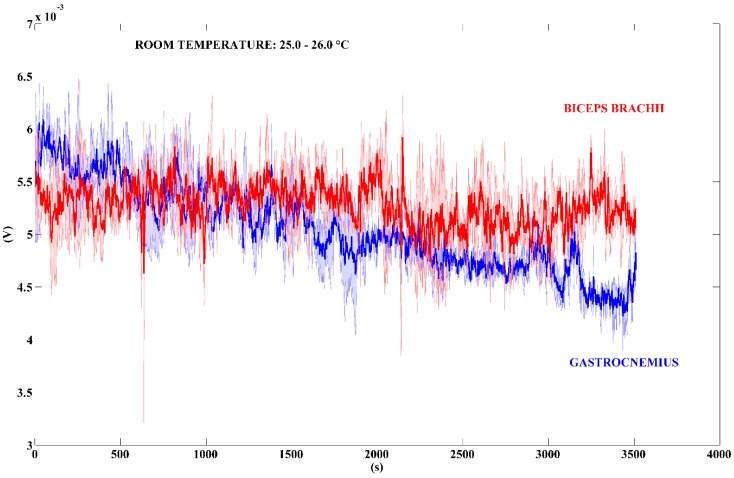
Voltage output signals generated by the TEG. The bold red line represents the mean value of the TEG placed on the biceps brachii; the bold blue line refers to the gastrocnemius. The blue and red areas around the bold lines represent the standard deviations (n = 5). Time-interval: 3500 s.

Working at the desk was the first activity performed in a real scenario. The TEG was placed on the biceps brachii and the gastrocnemius of the user. We monitored the TEG voltage signal for a week, from Monday to Friday. In this way, we collected five signals, in two different time-intervals, for both the mentioned muscles. In this scenario, the room temperature varies continuously due to the movement of people inside the office. Figure 9 shows the output voltage signals recorded in a time-interval of approximately 1350 s, while Figure 10 shows different voltage signals acquired for a longer time-interval of approximately 3500 s.

Table 3 summarizes the values of power output calculated by using the voltage data shown in Figure 9 and Figure 10. We used the MATLAB boxplot function to find out the mean power values related to the median, the 25th, and the 75th percentile values. The 25th percentile is the middle value between the smallest value and the median, while the 75th one is the middle value between the median and the highest one.

At first, it is important to denote that for a shortest time-interval monitoring, i.e., 1350 s (Figure 9), the mean power generated by the gastrocnemius muscle (6.18 µW) is higher than the power generated by the biceps brachii (3.92 µW). However, while analysing the longest period (Figure 10), i.e., 3500 s, it is easy to note that the output signal related to gastrocnemius muscle decreases at the end of the test: the mean power value is 3.92 µW. Conversely, the value of the output signal related to the biceps brachii muscle increase on the time: the mean power is 4.65 µW. That is due to the performed activity, in which the legs are in a steady position while the arms are moving during the work at the desk. Moreover, we made a further voltage measurement to show the decrease of the TEG output signal, acquired on the gastrocnemius muscle, in a time-interval of approximately 12,000 s (see Supplementary Materials, Figure S7).

**Figure 11:**
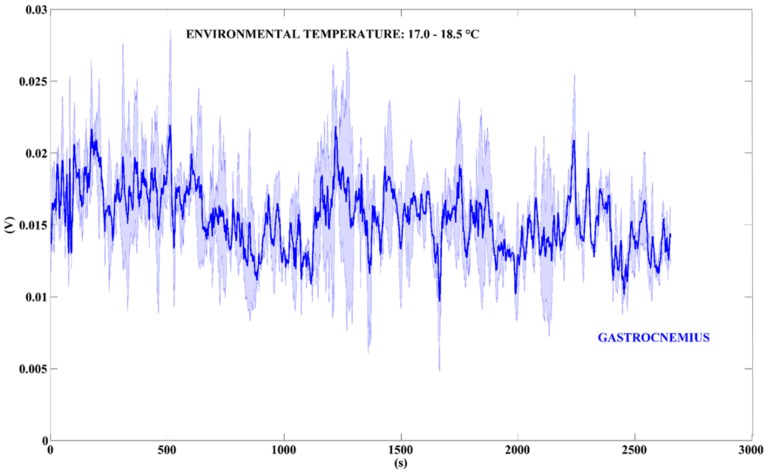
Voltage output signals generated by the TEG. The bold blue line represents the mean value of the TEG placed on the gastrocnemius. The blue area around the bold lines represents the standard deviations (n = 2). Time-interval: 2650 s.

Again, we monitored the voltage output signal of the TEG while the user was riding a bike (see Supplementary Materials, Figure S8). In this test, we decided to monitor only the signal acquired by placing the TEG on the gastrocnemius, since this activity involves the lower limbs much more than the upper ones. Figure 11 shows the output voltage signal recorded in a time-interval of approximately 2650 s.

Table 4 summarizes the values of power output calculated by using the voltage data shown in Figure 11, reporting the median, the 25th, and the 75th percentile values.

**Figure 12:**
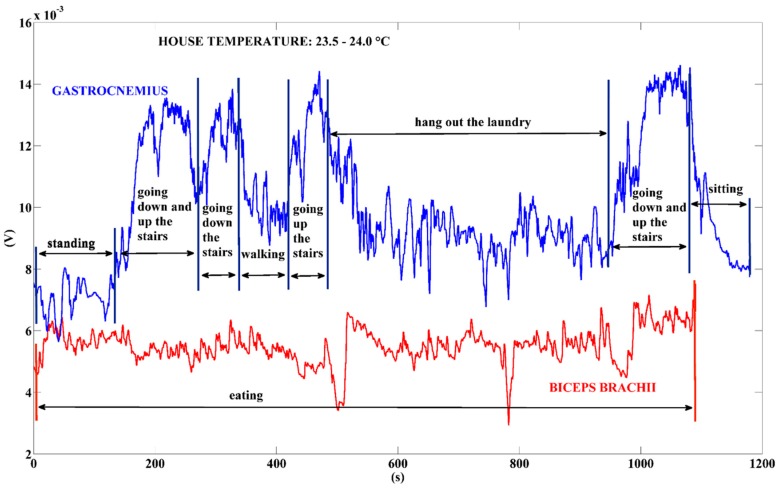
Voltage output signals generated by the TEG. The bold red line represents the voltage of the TEG placed on the biceps brachii while the user was eating; the bold blue line refers to the signal generated by the gastrocnemius muscle while the user was performing usual home activities. Time-interval: <1200 s.

While riding a bike the mean power output is 37.07 µW, with peaks of power values of approximately 50 µW, i.e., the 75th percentile value. Finally, Figure 12 shows the output signals of the TEG when the user is performing usual activities at home.

The mean power values corresponding to the voltage signals of Figure 12 are as follows: 16.23 µW for the power generated by the gastrocnemius muscle while the user is performing multiple activities; 5.34 µW for the power generated by the biceps brachii muscle while the user is eating. These preliminary measurements indicate the TEG placement on the leg as the best location to harvest the wasted human heat energy, especially when the user is performing locomotion activities. 

## 4. Discussion

During the performance of human daily activities, data collected in preliminary measurements suggested the use of a resistor load in the range from 4 to 6 Ω. In addition, the skin-area related to the biceps brachii muscle and to the gastrocnemius muscle resulted the best body-locations to harvest the thermal energy from arm and leg, respectively.

By using the proposed system, the temperature difference between the two sides of TEG has never exceeded 2.5 °C when the room temperature was approximately 25 °C. The developed band, made of PVC and gauze fabrics guaranteed softness and foldability but did not perfectly thermally insulated the two sides of TEG, as it was apparent from the decrease of temperature differences in graphs of the first experimental stage. However, the fabric band ensured comfort for users.

In the second and third stages of the experiment, data were collected during the execution of human activities, such as sitting, walking, jogging, working at desk, and riding a bike. The values of harvested power were in the range from 5 to 50 µW, thus confirming the possibility to use the TES1-12704 module for supplying energy to ultra-low power integrated circuits (ICs), such as nanopower operational amplifiers, temperature sensors, accelerometers and ICs used for the near field communication, among others [39,40,41].

The power values obtained in this work are in line with results of current literature regarding comfortable wearable system for harvesting body thermal energies. For instance, Hyland et al. [42] tested the behavior of a TEG placed on the upper arm at different walking speeds. At a walking speed of about 1.1 m/s, the TEG generates up to 20 µW/cm^2^. Again, Wahbah et al. [43] mounted a TEG with a small heatsink on a human wrist at room temperature of 22 °C; the maximum power output was equal to 20 µW.

Regarding TEGs not commercialized yet, researchers all over the world are proposing prototypes, in which flexible, or semi-foldable polymeric structures incorporate rigid thermoelectric elements [44,45,46,47]. The values of power output reach tens of microwatts only when temperature differences, between hot and cold side, exceed at least 10 °C. Unfortunately, it is very difficult to obtain a temperature difference higher than 10 °C between the skin and the environment, nowadays. Again, works presented by Siddique et al. [48] and Lu et al. [49] proposed TEG modules based on silk fabric and polyester fiber-cloth. These systems are more comfortable than the solution proposed here, since they are ultra-thin and foldable. However, by placing these TEG-fabrics on arm, the power output values do not exceed tens of nano-watts. 

Therefore, commercial TEGs are still rigid, since TEGs based on fabric, or semi-flexible structures do not guarantee sufficient generation of electrical energy to power commercial-off-the-shelf components.

The performed test while executing activities in a controlled environment, and in a real scenario lead to the hypothesis that the power harvested on the leg may represent a benefit to the results of the scientific literature, which are almost exclusively describing experiments of thermal energy harvesting on the upper body parts [50]. While analyzing the possibility to recognize daily activities based on the obtained power values, the results show that sitting activity is clearly distinguishable from the others, for both the TEG placement on biceps brachii and gastrocnemius muscles. Instead, for walking and jogging activities, the TEG placement on the biceps brachii generates similar values of power output, so it is hard to detect differences between walking and jogging. Conversely, the TEG placed on the gastrocnemius muscle generates different quantities of electrical power if the user is walking, jogging, or riding a bike. It is due to the gastrocnemius biomechanical work while performing these activities. By analyzing the results obtained in this work, we suggest the use of the TEG on the lower part of the body: the power values related to the gastrocnemius muscles are the highest, reaching values up to 50 µW.

By the results obtained in this work, a further medical application may be the use of TEGs to monitor a rehabilitation process of people with severe leg injuries. 

The TEG voltage output for a motionless person will be the reference value, and any improvement in locomotor activity would result in an increase of the TEG output value. However, the environmental temperature and physiological conditions should be kept constant throughout the rehabilitation process. 

Anyway, it would be useful to carry out further tests to confirm the preliminary hypothesis of using a TEG on leg to detect locomotion activities. Nowadays, the only way to recognize human activities through the use of energy harvesters is by exploiting the physical principle of kinetics [51]. 

## 5. Conclusions

The proposed paper investigates the performance of the TES1-12704 module for harvesting thermal energy directly on performing these activities, the power output was in the range from 5 to 50 µW. The placement of the TEG on the leg can also help to recognize locomotion activities, since leg temperature, as the thermal output of muscular work, can be directly associated with different locomotion activities. The results obtained in this paper are suggesting the energy harvesting, from the wasted heat of leg, may represent a new designing trend for developing wearable smart devices useful in a variety of applications, such as people’s safety, e-/m-health, telemedicine, rehabilitation, and wellness.

## Figures and Tables

**Table 1:** Skin temperature for different body positions.

Body Positions	Yang et al. [16] (T_air_ = 17 °C)	Zaproudina et al. [17] (T_air_ = 23.5 °C)	Webb [18] (T_air_ = 27 °C)
Forehead	29.5 °C	34.1 °C	35.2 °C
Neck	31.1 °C	33.2 °C	35.1 °C
Back	30.6 °C	32.5 °C	34.4 °C
Chest	30.3 °C	32.3 °C	34.4 °C
Arm anterior	30.3 °C	31.7 °C	33.2 °C
Forearm	29.5 °C	31.5 °C	34.0 °C
Thigh	28.3 °C	30.8 °C	33.0 °C
Calf	29.4 °C	31.3 °C	31.6 °C
Foot dorsal	27.1 °C	28.6 °C	30.4 °C

**Table 2:** Properties of the TES1-12704 module.

Property	Value
Dimension, (l × w × t)	30 mm × 30 mm × 3.2 mm
Weight	0.015 g
Ceramic substrate material	Aluminum oxide (Al_2_O_3_)
Metal conductors material	Copper (Cu)
Number of p-n couples	127
p- and n-type elements	Bismuth telluride (Bi_2_Te_3_)
Electrical conductivity, σ	800–13,501/(cm·Ω)
Thermal conductivity, κ	0.016–0.02 W/(cm·K)
Seebeck coefficient, α	160–200 µV/K
Coefficient of merit, Z	0.002695–0.0031/K

**Table 3:** Mean power values (n = 5). Working at desk activity.

Time-Interval (s)	Gastrocnemius Values (μW)			Biceps Brachii Values (μW)		
	25th	Median	75th	25th	Median	75th
1350	5.86 ± 0.92	6.18 ± 0.97	6.65 ± 1.04	3.44 ± 0.76	3.92 ± 1.09	4.47 ± 1.47
3500	3.52 ± 0.24	4.01 ± 0.26	4.67 ± 0.34	4.24 ± 0.18	4.65 ± 0.13	5.03 ± 0.24

**Table 4:** Mean power values (n = 2). Working at desk activity.

Time-Interval (s)	Gastrocnemius Values (μW)		
	25th	Median	75th
2650	27.77 ± 1.20	37.07 ± 0.40	51.79 ± 1.42

## Supplementary Materials

The following are available online at http://www.mdpi.com/1424-8220/18/6/1927/s1. Figure S1: TEG measuring system. Figure S2: NTC 10K3MBD1 thermistor probes on TEG upper side, and multichannel recording system. Figure S3: Chosen materials: gauze and PVC fabrics. Figure S4: Developed band for thermal body energy harvesting. Figure S5: Placement of TEG on upper body parts. Figure S6: Placement of TEG on lower body parts. Figure S7: TEG voltage output when leg remains in a steady position for long time (11,000 s). Figure S8: User riding a bike while wearing the TEG.
