# Supplementary material for: Thermal Energy Harvesting on the Bodily Surfaces of Arms and Legs through a Wearable Thermo-Electric Generator
Source: Sensors (Basel). 2018 Jun 13;18(6):1927. doi: 10.3390/s18061927 (PMC6022077; doi:10.3390/s18061927)
Supplement: Supplementary file 1 [file sensors-18-01927-s001.pdf]

sensors

MDPI

Supplementary Materials

Thermal Energy Harvesting on the Bodily Surfaces of Arms and Legs through a Wearable Thermo-Electric Generator

Antonino Proto [1], Daniele Bibbo [2], Martin Cerny [1], David Vala [1], Vladimir Kasik [1], Lukas Peter [1], Silvia Conforto [2], Maurizio Schmid [2] and Marek Penhaker [1,*]

[1] VŠB-Technical University of Ostrava, Department of Cybernetics and Biomedical Engineering, 17. listopadu 15, Ostrava-Poruba 70833, Czech Republic; antonino.proto@vsb.cz (A.P); martin.cerny@vsb.cz (M.C.); david.vala@vsb.cz (D.V.); vladimir.kasik@vsb.cz (V.K.); lukas.peter@vsb.cz (L.P.).

[2] University of Roma Tre, Department of Engineering, Via Vito Volterra, 62, Rome 00146, Italy; daniele.bibbo@uniroma3.it (D.B.); silvia.conforto@uniroma3.it (S.C.); maurizio.schmid@uniroma3.it (M.S.).

* Correspondence: marek.penhaker@vsb.cz (M.P.); Tel.: +420-897-325-853

Figure S1 shows the chain of the system for measurements the TEG voltage output.

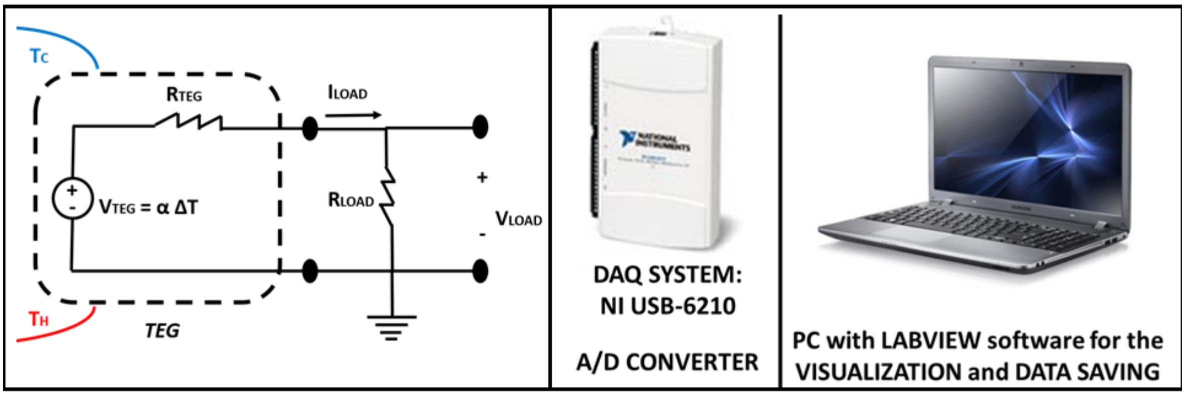

Figure S1. TEG measuring system.

Figure S2 shows the placement of the NTC 10K3MBD1 thermistor probes on the upper side of the TEG, and the multichannel recording system.

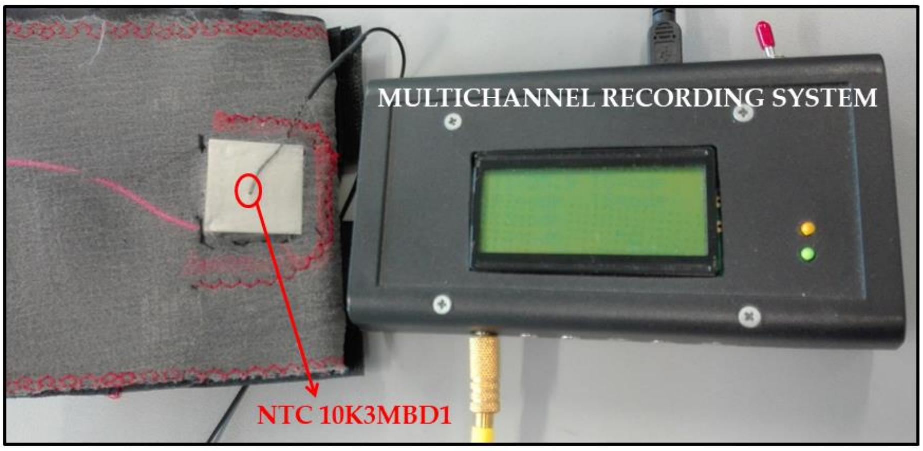

Figure S2. NTC 10K3MBD1 thermistor probes on TEG upper side, and multichannel recording system.

Figures S3 and S4 show the chosen materials, and the developed band, respectively.

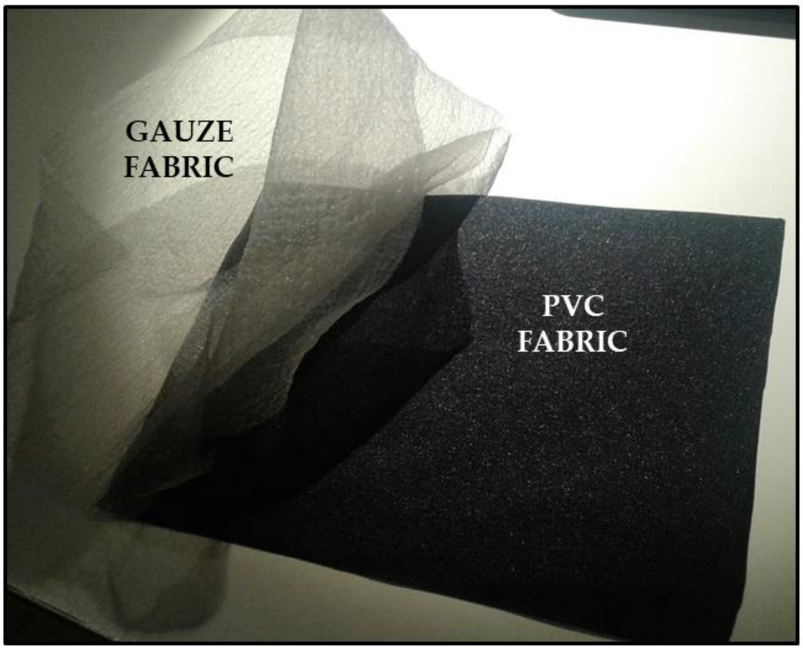

Figure S3. Chosen materials: gauze and PVC fabrics.

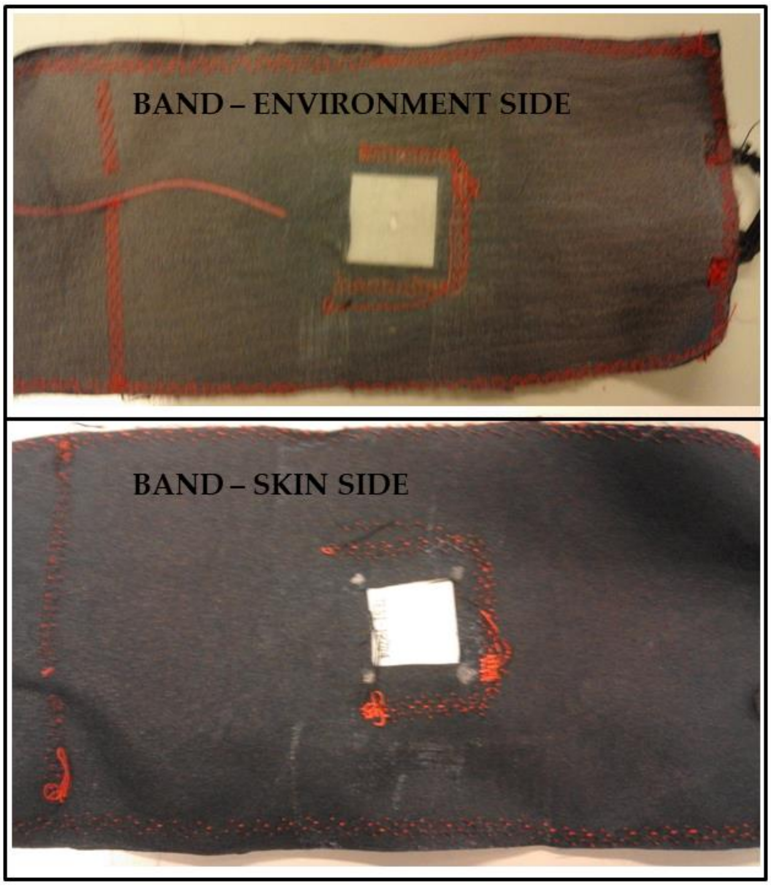

Figure S4. Developed band for thermal body energy harvesting.

Biceps brachii for the arm anterior and flexor carpi radialis for the forearm were the muscles corresponding to the placement of the TEG on the upper body part (Figure S5), while gracilis for the

thigh and gastrocnemius for the calf were the chosen muscles for placing the TEG on the leg (Figure S6).

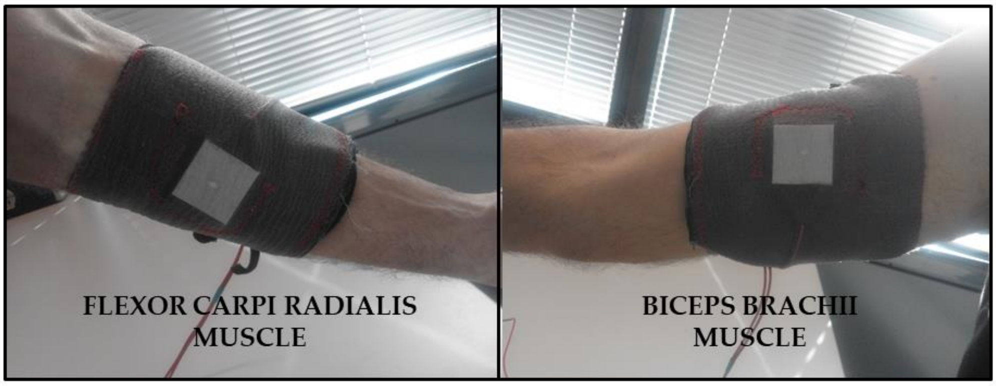

Figure S5. Placement of TEG on upper body parts.

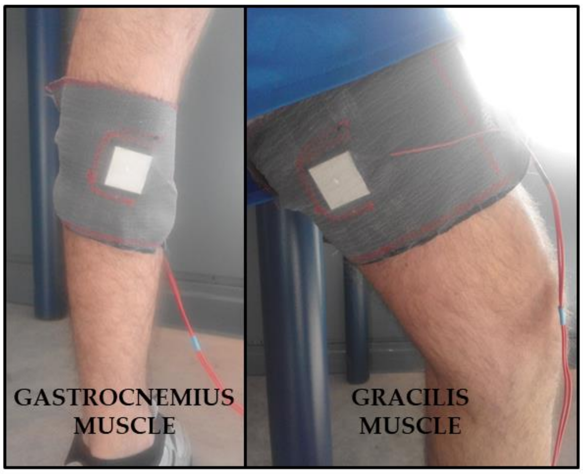

Figure S6. Placement of TEG on lower body parts.

Figure S7 shows a voltage measurement to show the decrease of the TEG output signal, acquired on the gastrocnemius muscle, in a time-interval of approximately 11,000 s.

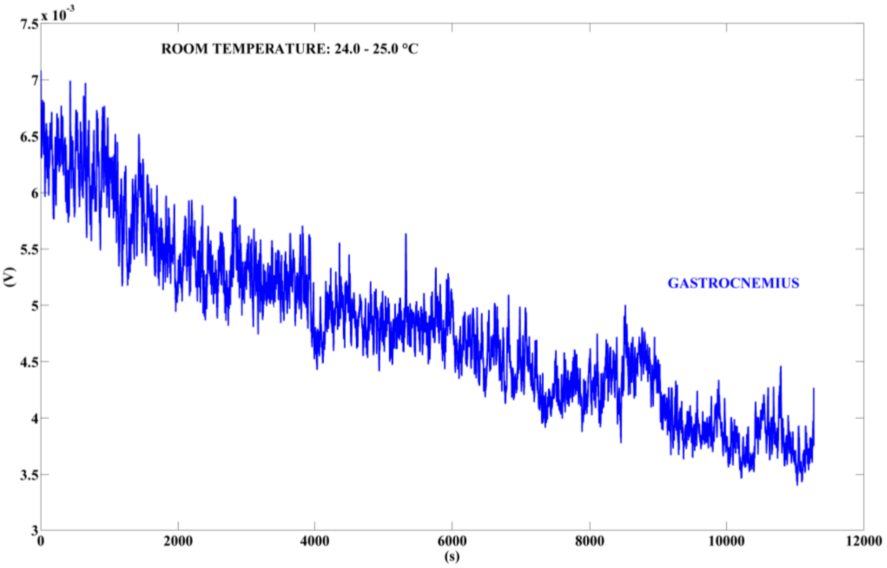

Figure S7. TEG voltage output when leg remains in a steady position for long time (11,000 s).

Figure S8 shows the user riding a bike.

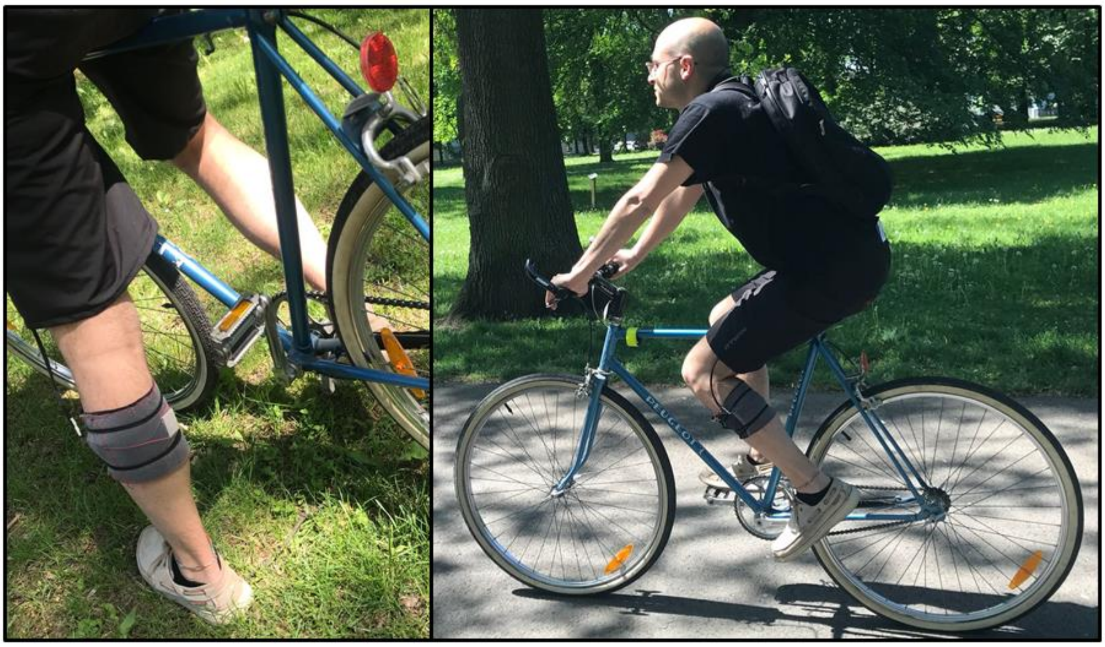

Figure S8. User riding a bike while wearing the TEG.
